# Neuropsychological measures of post-COVID-19 cognitive status

**DOI:** 10.3389/fpsyg.2023.1136667

**Published:** 2023-07-10

**Authors:** Alessandra Lauria, Angelo Carfì, Francesca Benvenuto, Giulia Bramato, Francesca Ciciarello, Sara Rocchi, Elisabetta Rota, Andrea Salerno, Leonardo Stella, Marcello Tritto, Antonella Di Paola, Cristina Pais, Matteo Tosato, Delfina Janiri, Gabriele Sani, Rita Lo Monaco, Francesco C. Pagano, Massimo Fantoni, Roberto Bernabei, Francesco Landi, Alessandra Bizzarro, Gemelli Against COVID-19 Post-acute Care Group

**Affiliations:** Gemelli Against COVID-19 Post-acute Care Study Group, Fondazione Policlinico Universitario Agostino Gemelli IRCCS, Rome, Italy

**Keywords:** COVID-19, neuropsychological assessment, Foggy Brain, long COVID, cognitive evaluation

## Abstract

**Background:**

COVID-19 may result in persistent symptoms in the post-acute phase, including cognitive and neurological ones. The aim of this study is to investigate the cognitive and neurological features of patients with a confirmed diagnosis of COVID-19 evaluated in the post-acute phase through a direct neuropsychological evaluation.

**Methods:**

Individuals recovering from COVID-19 were assessed in an out-patient practice with a complete neurological evaluation and neuropsychological tests (Mini-Mental State Examination; Rey Auditory Verbal Test, Multiple Feature Target Cancellation Test, Trial Making Test, Digit Span Forward and Backward, and Frontal Assessment Battery). Pre- and post-COVID-19 global and mental health status was assessed along with the history of the acute phase of infection. Post-COVID-19 cognitive status was modeled by combining persistent self-reported COVID-related cognitive symptoms and pathologic neuropsychological tests.

**Results:**

A total of 406 individuals (average age 54.5 ± 15.1 years, 45.1% women) were assessed on average at 97.8 ± 48.0 days since symptom onset. Persistent self-reported neurological symptoms were found in the areas of sleep (32%), attention (31%), and memory (22%). The MMSE mean score was 28.6. In total, 84 subjects (20.7%) achieved pathologic neuropsychological test results. A high prevalence of failed tests was found in digit span backward (18.7%), trail making (26.6%), and frontal assessment battery (10.9%). Cognitive status was associated with a number of factors including cardiovascular disease history, persistent fatigue, female sex, age, anxiety, and mental health stress.

**Conclusion:**

COVID-19 is capable of eliciting persistent measurable neurocognitive alterations particularly relevant in the areas of attention and working memory. These neurocognitive disorders have been associated with some potentially treatable factors and others that may stratify risk at an early stage.

## What is already known on this topic

COVID-19 is known to cause several persistent symptoms during the recovery phase, including cognitive and neurological symptoms that can go on for a long time.

## What this study adds

COVID-19 can elicit persistent measurable neurocognitive alterations particularly relevant in the areas of attention and working memory. These neurocognitive disorders were associated with several factors: some potentially treatable factors and other factors capable of early risk stratification.

## How this study might affect research, practice, or policy

After COVID-19, patients could benefit from a neurologic and cognitive assessment. Those with pathologic neuropsychological test results should be monitored over time to understand whether the infection can be the fuse of latent dementia.

## Introduction

Since December 2019, when the outbreak of coronavirus infectious disease 2019 (COVID-19) was first identified in China's Hubei region, the pandemic of severe acute respiratory syndrome coronavirus 2 (SARS-CoV-2) has now spread throughout the world, impacting populations and health systems globally. Documentation of the long-term effects of COVID-19 includes fatigue, shortness of breath, pain (Carfì et al., 2020; Nalbandian et al., 2021) as well as neurological involvement and psychological symptoms affecting as much as one-third of infected people (Huang et al., 2021; Janiri et al., 2021; Taquet et al., 2021).

Both during the acute phase and after recovery, neuropsychological manifestations are very diverse and may include anxiety, mood alterations, forgetfulness, trouble focusing, and a general sense of mental sluggishness, often referred to as “Covid Fog” or “Foggy Brain” (Carfì et al., 2020; Graham et al., 2021; Huang et al., 2021; Janiri et al., 2021; Nalbandian et al., 2021; Taquet et al., 2021). These symptoms can affect multiple dimensions of daily life, such as physical and social well-being and work performance (Tenforde et al., 2020). Some research ascribed a number of the cognitive disorders to a diagnosis of dementia (Taquet et al., 2021).

The mechanisms by which SARS-CoV-2 interacts with the nervous system are not yet fully clarified (Iadecola et al., 2020). Viral neurotropism is so far poorly supported by biological studies, a fact also related to the low expression in the central nervous system (CNS) of the canonical set of receptors associated with viral infection. Although multiple pathways into the CNS are hypothesized (the olfactory pathway, through the blood-brain barrier or infiltration by infected immune cells), clinical and autopsy data have not been able to conclusively demonstrate direct invasion of the central nervous system by SARS-CoV-2. Conversely, the indirect effect on the brain of a complex interplay of systemic factors could play a key role in the pathogenesis of neurological symptoms. These factors include both systemic and local CNS cytokine release. COVID-19 is notoriously associated with high circulating levels of cytokines. At the same time, local CNS cytokine release is also hypothesized to activate when triggered by circulating viral and cellular debris on the innate response cells such as vascular pericytes and brain resident macrophages and microglia. Hypoxia, endothelial damage, dysfunction in blood pressure regulation and intracerebral hemorrhage have also been accounted as important players in the pathogenesis of these disturbances.

The incredibly heterogeneous clinical manifestations, including psychological distress, organic disease and their intermixture, together with the many possible pathophysiological and neuropsychological mechanisms involved in their pathogenesis, make the study of neurological involvement in COVID-19 particularly complex (Ritchie et al., 2020; Finsterer and Scorza, 2021) and are at least partly responsible for the relative paucity of available data. Moreover, the available results often suffer from the limitation of being based on indirect measures such as in studies reviewing medical records or in studies relying solely on patient self-report (Graham et al., 2021; Taquet et al., 2021). Based on this premises, this study aimed to use a direct neuropsychological assessment to examine the cognitive and neurologic features of a group of people with a confirmed diagnosis of COVID-19 evaluated in the post-acute phase.

## Methods

Since the end of April 2020, our institution has started an outpatient service dedicated to the evaluation and treatment of individuals healed from COVID-19 in the convalescent phase. Inclusion criteria were the previous diagnosis of COVID-19 confirmed by a nasopharyngeal swab and meeting the criteria for quarantine discontinuation (no fever, improvement of other symptoms, and 2 nasopharyngeal swabs tested negative 24 hours apart).

Initially the service enrolled consecutive patients who had received acute phase care at our hospital as the clinic staff contacted them to offer the possibility to be followed at our service. At a later time, patients from the territory also turned to the service in order to be evaluated.

Each enrolled subject received a number of assessments [described elsewhere (Gemelli Against COVID-19 Post-Acute Care Study Group, 2020)] which included a detailed medical history, neurological physical examination, and a history specific to general and neurological symptoms related both to the acute phase of COVID-19 and to the recovery phase at the time of evaluation. For this purpose, a specific questionnaire was designed for pre and post COVID-19 neurological symptoms. Pre-existence or non-existence of neurological disorders in the period prior to the coronavirus infection was recorded.

### Cognitive evaluation

The neuropsychological evaluation was administered using a specially constructed battery to meet both the accuracy and completeness needed for the most thorough assessment possible and the fast pace requirements of the clinical activities. Together with the memory domains, in consideration of the involvement of subcortical structures in other pathologies with viral [e.g., HIV neuro-cognitive disorder (Clifford and Ances, 2013)] or immune-mediated [e.g., multiple sclerosis (Rovaris et al., 2000)] etiology, a number of neuropsychological tests capable of accurately study executive functions were chosen.

For each subject, the cognitive assessment was performed with Mini Mental State Examination (Folstein et al., 1975) and the following 8 tests: Rey Auditory Verbal Test was used to evaluate immediate and deferred memory (Carlesimo et al., 1995); Multiple Features Target Cancellation Test to assess visual-spatial exploration and selective attention (Gainotti et al., 2001); Trial Making Test (B-A score) assessed selective, divided and alternating, attention, inhibition control and working memory (Bowie and Harvey, 2006); Digit span Forward and Backward evaluated working and verbal short-term memory (Monaco et al., 2013); Frontal Assessment Battery evaluates composite multidimensional domains and was used to screen for global executive dysfunction including cognitive, affective, behavioral and motivational components (Appollonio et al., 2005).

For each neuropsychological test, the raw scores, the scores adjusted for age and educational level and gender (where appropriate), and standardized scores on a 5-point ordinal scale (Equivalent Scores, ES) (Capitani and Laiacona, 1997) were reported. Equivalent scores served a double purpose. On one hand they allowed to coherently define individuals with impaired performance. On the other hand, helped to accurately compare the performances from the various tests in order to obtain a cognitive profile for each subject. A test score of 0 was considered pathological, a score of 1 was classified as borderline, and scores of 2 to 4 were considered consistent with normal performance.

### Outcome definitions

Due to the unavailability of pre-pandemic neuropsychological tests of the examined subjects, in order to simultaneously describe both the neurocognitive effect possibly attributable to COVID-19 and objective measurement derived from the tests, we defined two main entities. From the history, we categorized patients according to whether or not they presented with at least one persistent COVID-related neurologic symptom (PCNS) defined as either a symptom that began with COVID-19 and did not regress at the time of evaluation, or a symptom pre-existing at COVID-19 that was reported as persistently worsened until the time of assessment. From the neuropsychological tests, we classified patients with pathologic neuropsychological tests (PNT) based on whether they had scored at least one pathological and at least one borderline test. As sketched in the schematic below, we considered the study's primary outcome as the cognitive status described by the intersection of the two entities, i.e., by the following four categories: intact cognition, asymptomatic post-COVID cognitive disturbances, subjective persistent COVID-related cognitive sequelae, and persistent COVID-related cognitive disturbances.

**Table:** 

		**PNT Pathologic Neuropsychological Tests**	
		**No**	**Yes**
PCNS Persistent Covid-related Neurologic Symptoms	No	Intact cognition	APCCD Asymptomatic Post-Covid Cognitive Disturbances
	Yes	SPCCS Subjective Persistent Covid-related Cognitive Sequelae	PCCDPersistent Covid-related Cognitive Disturbances

### Other evaluations

Psychiatric features were assessed by a set of standardized scales: anxiety symptoms by the Hamilton Anxiety (Ham-A) (Maier et al., 1988), depressive symptoms by the Hamilton Depression (Ham-D) (Zimmerman et al., 2013), and global psychological distress by the Kessler Psychological Distress (K10) (Kessler et al., 2002). Cut-offs of 7, 7 and 19 in the total score of each scale respectively were used to indicate pathological scores (Maier et al., 1988; Kessler et al., 2002; Zimmerman et al., 2013). The Pittsburg Sleep Quality Index (PSQI) (Buysse et al., 1989) was used to assess sleep quality, and the cut-off of 5 was used to detect sleep disturbances. The seven-category ordinal scale (Cao et al., 2020) was used to stratify case severity. Based on the need for hospitalization and O2 administration it classifies subjects into: 1, not hospitalized with resumption of normal activities; 2, not hospitalized, but unable to resume normal activities; 3, hospitalized, not requiring supplemental oxygen; 4, hospitalized, requiring supplemental oxygen; 5, hospitalized, requiring nasal high-flow oxygen therapy, noninvasive mechanical ventilation, or both; 6, hospitalized, requiring invasive mechanical ventilation, Extra-Corporeal Membrane Oxygenation (ECMO), or both; and 7, death. In view of the fact that the assessment occurred several weeks after the acute event, categories 1 and 2 were regrouped into a single category to which score 2 was arbitrarily assigned. Given the differences in the clinical manifestations and severity between sexes (Scully et al., 2020), results were also compared by sex.

### Statistical analysis

Descriptive analyses and comparisons were obtained through ANOVA and χ^2^ tests where appropriate.

Multinomial logistic regression was used in a secondary exploratory analysis of the association between the cognitive outcome and a number of variables of importance including demographic factors, pre-Covid health status, acute and post-Covid parameters. In the logistic regression the missing values were managed with multiple imputation by chained equations (van Buuren and Groothuis-Oudshoorn, 2011) technique using random forest algorithm (Shah et al., 2014). Details on coding variables and handling missing data are provided in the Supplementary material.

The *p*-value was set to < 0.05 for statistical significance. Given the descriptive and exploratory basis of the analyses no correction of significance levels was used. All analyses were conducted using R version 4.1.3 (R Foundation).

This study was approved by the Università Cattolica del Sacro Cuore and Fondazione Policlinico Gemelli IRCCS Institutional Ethics Committee. Written informed consent was obtained from all participants.

## Results

We present data from 406 individuals (average age 54.5 ± 15.1 years, 45.1% women) assessed at our institution from April 23, 2020 to November 30, 2020.

The general characteristics of the study subjects, stratified by age and cognitive status, are described in Supplementary Table 1.

As in the general population, women presented with a lower prevalence of cardiovascular disease history and a higher prevalence of thyroid disorders. In contrast, men showed a more challenging course of COVID-19, characterized by more intensive medical care and longer length of stay. Nevertheless, they exhibited less persistence of post-COVID-19 symptoms and, on average, a smaller decrease in quality-of-life scores.

During the neurologist interview, a high prevalence of persistent COVID-19-related neurological symptoms (initiated during COVID-19 or pre-existing at COVID-19 and persistently worsened after that) was evident particularly in the areas of sleep (32%), attention (30%), and memory (22%) as detailed in the Supplementary Table 2 and Supplementary Figures 1, 2. Notably, 22% of subjects reported suffering from at least one of the investigated neurological symptoms already before COVID-19, and 47% reported, following COVID-19, either the persistence of at least one new-onset neurological symptom or the worsening of a pre-existing neurological symptom.

The outcome of the neuropsychological tests is shown in Table 1, Supplementary Table 3, and Supplementary Figure 3. The overall mean MMSE score was 28.6, as expected in a study sample of relatively young, cognitively intact individuals. Differences between the two sexes and age groups reached statistical significance; however, as these were small variations largely within normal ranges, no clinical significance was attributed to them. The cross-counting of neuropsychological test results for each individual showed that 84 subjects (20.7%) had pathological scores with at least one failed test and at least one borderline test.

**Table 1:** Neuropsychological tests.

	**Sex**				**Age groups**			
	**Total**	**Males**	**Females**	***p***	**Under 44**	**45–64**	**65 or more**	***p***
	***n*** = **406**	***n*** = **223**	***n*** = **183**		***n*** = **93**	***n*** = **213**	***n*** = **100**	
**MMSE**								
Corrected	28.6 (1.6)	28.7 (1.5)	28.4 (1.7)	**0.028**	28.9 (1.8)	28.6 (1.6)	28.3 (1.5)	**0.027**
**Rey's immediate recall**								
Corrected	45.5 (8.3)	45.1 (8.8)	45.9 (7.6)	0.346	47.1 (7.2)	46.1 (8.6)	42.6 (7.8)	**< 0.001**
Equivalent	3.5 (1.0)	3.4 (1.1)	3.6 (0.9)	**0.031**	3.7 (0.7)	3.5 (1.0)	3.1 (1.1)	**< 0.001**
Failed	9 (2.2%)	6 (2.7%)	3 (1.6%)		0 (0%)	7 (3.3%)	2 (2%)	
Borderline	17 (4.2%)	13 (5.8%)	4 (2.2%)		2 (2.2%)	6 (2.8%)	9 (9%)	
**Rey's delayed recall**								
Corrected	9.5 (2.7)	9.2 (2.8)	9.9 (2.4)	**0.011**	10.2 (2.7)	9.6 (2.4)	8.7 (2.9)	**< 0.001**
Equivalent	3.3 (1.0)	3.2 (1.1)	3.5 (0.9)	**< 0.001**	3.6 (0.7)	3.4 (0.9)	3.0 (1.3)	**< 0.001**
Failed	11 (2.7%)	8 (3.6%)	3 (1.6%)		0 (0%)	4 (1.9%)	7 (7%)	
Borderline	18 (4.4%)	11 (4.9%)	7 (3.8%)		2 (2.2%)	6 (2.8%)	10 (10%)	
**MFTC Test**								
*Time* corrected	53.8 (22.6)	56.3 (20.6)	50.8 (24.5)	**0.015**	59.4 (19.0)	52.8 (18.1)	50.8 (31.7)	**0.01**
*Time* equivalent	3.9 (0.5)	3.9 (0.5)	3.9 (0.6)	0.919	3.8 (0.6)	3.9 (0.4)	3.8 (0.8)	**0.043**
Failed	3 (0.7%)	1 (0.4%)	2 (1.1%)		1 (1.1%)	0 (0%)	2 (2%)	
Borderline	3 (0.7%)	1 (0.4%)	2 (1.1%)		0 (0%)	1 (0.5%)	2 (2%)	
*False alarms* corrected	0.5 (1.8)	0.5 (2.1)	0.5 (1.3)	0.955	0.4 (1.5)	0.4 (1.5)	0.7 (2.5)	0.54
*False alarms* equivalent	3.5 (1.1)	3.6 (1.0)	3.5 (1.1)	0.098	3.5 (1.0)	3.6 (1.0)	3.5 (1.1)	0.881
Failed	16 (3.9%)	8 (3.6%)	8 (4.4%)		2 (2.2%)	7 (3.3%)	7 (7%)	
Borderline	17 (4.2%)	6 (2.7%)	11 (6%)		5 (5.4%)	10 (4.7%)	2 (2%)	
**Frontal assessment battery**								
Corrected	16.1 (1.6)	16.2 (1.7)	16.1 (1.6)	0.958	16.4 (1.4)	16.2 (1.6)	15.8 (1.9)	0.059
Equivalent	3.0 (1.1)	3.0 (1.1)	2.9 (1.1)	0.43	3.1 (1.0)	3.0 (1.1)	2.8 (1.4)	0.299
Failed	23 (5.7%)	11 (4.9%)	12 (6.6%)		2 (2.2%)	9 (4.2%)	12 (12%)	
Borderline	21 (5.2%)	14 (6.3%)	7 (3.8%)		3 (3.2%)	10 (4.7%)	8 (8%)	
**Digit span forward**								
Corrected	5.8 (1.2)	6.0 (1.2)	5.7 (1.2)	**0.047**	5.8 (1.2)	5.8 (1.2)	6.0 (1.1)	0.359
Equivalent	3.1 (1.2)	3.1 (1.2)	3.0 (1.2)	0.177	3.0 (1.1)	3.0 (1.3)	3.2 (1.3)	0.555
Failed	33 (8.1%)	19 (8.5%)	14 (7.7%)		4 (4.3%)	21 (9.9%)	8 (8%)	
Borderline	11 (2.7%)	4 (1.8%)	7 (3.8%)		7 (7.5%)	0 (0%)	4 (4%)	
**Digit span backwards**								
Corrected	4.0 (1.0)	4.2 (1.0)	3.8 (0.9)	**< 0.001**	3.9 (1.0)	4.0 (1.0)	4.1 (1.0)	0.437
Equivalent	2.7 (1.3)	2.8 (1.2)	2.4 (1.3)	**0.002**	2.5 (1.4)	2.7 (1.2)	2.7 (1.3)	0.42
Failed	32 (7.9%)	12 (5.4%)	20 (10.9%)		11 (11.8%)	13 (6.1%)	8 (8%)	
Borderline	44 (10.8%)	24 (10.8%)	20 (10.9%)		8 (8.6%)	21 (9.9%)	15 (15%)	
**Trail making**								
Corrected	100.8 (71.4)	89.6 (68.5)	114.3 (72.7)	**< 0.001**	107.8 (50.6)	89.5 (60.2)	118.2 (100.6)	**0.003**
Equivalent	2.5 (1.3)	2.7 (1.3)	2.2 (1.3)	**< 0.001**	2.1 (1.1)	2.7 (1.2)	2.3 (1.5)	**< 0.001**
Failed	38 (9.4%)	17 (7.6%)	21 (11.5%)		4 (4.3%)	13 (6.1%)	21 (21%)	
Borderline	70 (17.2%)	28 (12.6%)	42 (23%)		30 (32.3%)	29 (13.6%)	11 (11%)	
**Other measures**								
PSQI	7.7 (4.1)	6.5 (3.4)	9.2 (4.4)	**< 0.001**	7.6 (4.1)	7.8 (4.2)	7.6 (4.0)	0.921
PSQI > 5	262 (64.5%)	122 (54.7%)	140 (76.5%)	**< 0.001**	57 (61.3%)	135 (63.4%)	70 (70%)	0.333
Ham-A	6.7 (6.7)	4.9 (5.5)	8.8 (7.4)	**< 0.001**	7.0 (6.5)	7.5 (7.2)	4.8 (5.5)	**0.005**
Ham-A > 7	131 (32.3%)	49 (22%)	82 (44.8%)	**< 0.001**	38 (40.9%)	75 (35.2%)	18 (18%)	**< 0.001**
Ham-D	5.0 (4.7)	4.0 (4.3)	6.2 (4.9)	**< 0.001**	4.9 (4.4)	5.7 (5.0)	3.7 (4.1)	**0.002**
Ham-D > 7	104 (25.6%)	44 (19.7%)	60 (32.8%)	**0.004**	27 (29%)	61 (28.6%)	16 (16%)	**0.022**
K10	17.8 (6.9)	16.0 (6.4)	19.8 (7.0)	**< 0.001**	19.2 (8.0)	18.1 (6.8)	15.5 (5.4)	**0.003**
K10 > 19	107 (26.4%)	41 (18.4%)	66 (36.1%)	**< 0.001**	30 (32.3%)	62 (29.1%)	15 (15%)	**0.039**

Values are expressed as mean (SD) for numeric variables and count (%) for categorical variables.

MMSE, Mini-Mental State Exam; MFTC, Multiple Features Target Cancellation test; PSQI, Pittsburg Sleep Quality Index; Ham-A, Hamilton Anxiety scale; Ham-D, Hamilton Depression scale; K10, Kessler Psychological Distress.

Values in bold highlight statistically significant differences.

It is important to note that 10.9, 18.7, and 26.6% of the subjects achieved either pathological or borderline performance in the Frontal Assessment Battery, Digit Span Backwards, and Trail Making test, respectively.

Importantly, 64.5% of subjects presented PSQI scores consistent with sleep disorder, 32.3% presented Ham-A scale scores consistent with anxiety symptoms, 25.6% Ham-D scores consistent with depressive symptoms, and 26.4% K10 scores consistent with stress.

**Figure 1:**
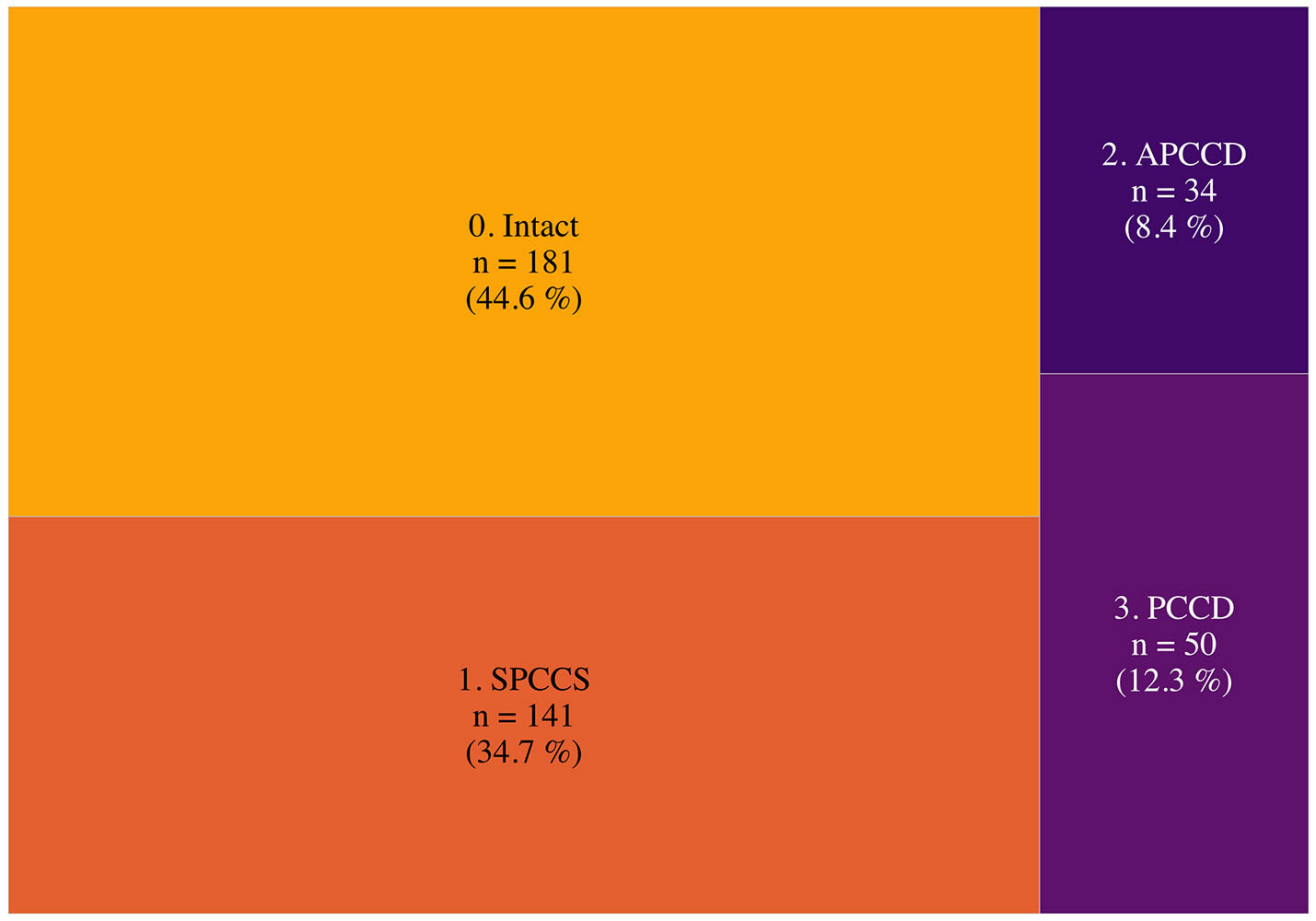
Post-COVID-19 cognitive status. This figure shows the relative frequencies of the post-COVID-19 cognitive status. Surface area is proportional to relative frequencies. APCCD, asymptomatic post-COVID cognitive disturbances (no persistent symptoms but pathologic NPS tests); SPCCS, subjective persistent COVID-related cognitive sequelae (persistent symptoms but normal NPS tests); PCCD, persistent COVID-related cognitive disturbances (persistent symptoms and pathologic NPS test).

Figure 1 shows how the study subjects were distributed across the four categories of cognitive status. It is important to note that less than half of the subjects fell into the intact cognition category. In total, 34.7% were classified as subjective persistent COVID-related cognitive sequelae (SPCCS), i.e., reporting persistent symptoms but scoring normal NPS tests, 12.3% as persistent COVID-related cognitive disturbances (PCCD), i.e., reporting persistent symptoms and scoring pathologic NPS tests, while 8.4% as asymptomatic post-COVID cognitive disturbances (APCCD), i.e., without persistent symptoms but with pathologic NPS tests.

**Figure 2:**
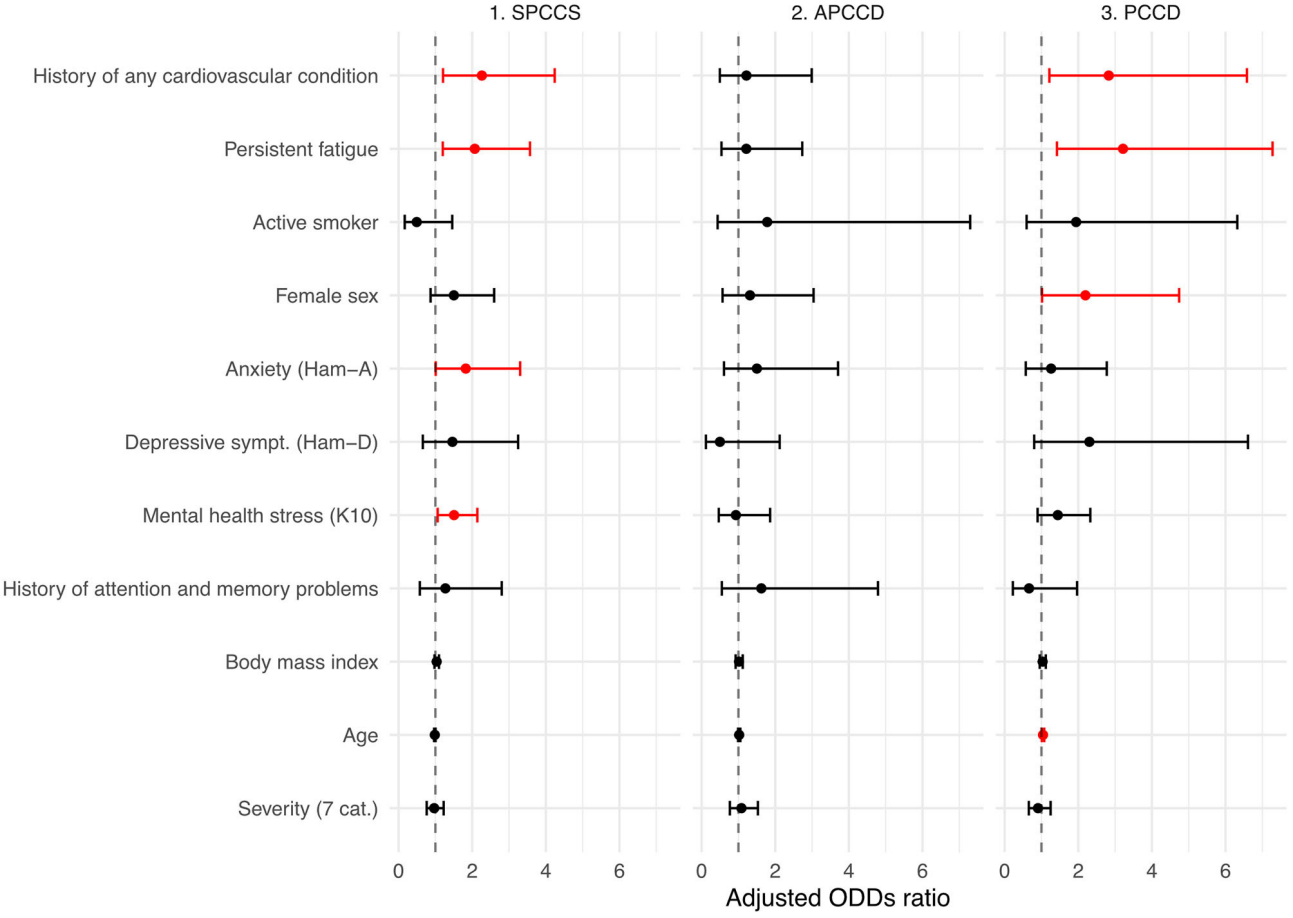
Factors associated with post-COVID-19 cognitive status. Adjusted odds ratios and 95% confidence intervals for the association between clinically relevant factors and post-COVID-19 cognitive outcomes. Intact cognition was set as the reference point. Red color means statistically significant association. APCCD, asymptomatic post-COVID cognitive disturbances (no persistent symptoms but pathologic NPS tests); SPCCS, subjective persistent COVID-related cognitive sequelae (persistent symptoms but normal NPS tests); PCCD, persistent COVID-related cognitive disturbances (persistent symptoms and pathologic NPS test); Ham-A, Hamilton anxiety scale; Ham-D, Hamilton depression scale; K10, Kessler Psychological Distress scale; Severity, Seven-category Ordinal Scale.

Table 2 and Figure 2 show the results of multinomial logistic regression in which the probability of being classified into one of the four cognitive status categories was associated with a set of variables of interest. The reference point was the intact category. A history of cardiovascular disease and persistent fatigue was found to be associated with being categorized as SPCCS or PCCD. Anxiety and mental health stress were associated with SPCCS, while sex and age were associated with PCCD. No factors were found to be significantly associated with the APCCD category. In addition, BMI, smoking status, and acute COVID-19 severity indicators were not found to be significantly associated with any of the categories.

**Table 2:** Multinomial logistic regression of post-COVID-19 cognitive status.

	**SPCCS**			**APCCD**			**PCCD**		
	**Adj.OR**	**95% C.I**.	***p***	**Adj.OR**	**95% C.I**.	***p***	**Adj.OR**	**95% C.I**.	***p***
Age	0.98	(0.96–1.01)	0.135	1.02	(0.99–1.05)	0.210	1.04	(1.01–1.07)	**0.009**
Female sex	1.50	(0.87–2.60)	0.145	1.31	(0.57–3.04)	0.522	2.19	(1.02–4.74)	**0.045**
Body mass index	1.03	(0.97–1.10)	0.261	1.02	(0.92–1.12)	0.741	1.03	(0.95–1.12)	0.434
Active smoker	0.49	(0.17–1.46)	0.200	1.78	(0.43–7.30)	0.421	1.94	(0.60–6.32)	0.270
History of attention and memory problems	1.27	(0.58–2.80)	0.551	1.62	(0.55–4.79)	0.380	0.66	(0.22–1.97)	0.458
History of any cardiovascular condition	2.26	(1.21–4.24)	**0.011**	1.22	(0.50–2.99)	0.667	2.83	(1.21–6.58)	**0.016**
Severity (7 categories)	0.97	(0.76–1.22)	0.779	1.08	(0.77–1.53)	0.650	0.91	(0.66–1.25)	0.546
Persistent fatigue	2.07	(1.20–3.57)	**0.009**	1.21	(0.54–2.73)	0.639	3.22	(1.42–7.28)	**0.005**
Anxiety (Ham–A)	1.83	(1.01–3.30)	**0.047**	1.50	(0.61–3.71)	0.377	1.26	(0.57–2.77)	0.561
Depressive symptoms (Ham–D)	1.46	(0.66–3.25)	0.349	0.50	(0.12–2.12)	0.344	2.30	(0.80–6.61)	0.121
Mental health stress (K10)	1.51	(1.06–2.14)	**0.022**	0.93	(0.47–1.86)	0.837	1.44	(0.90–2.33)	0.130

Adjusted odds ratios and 95% confidence intervals for the association between clinically relevant factors and post-COVID-19 cognitive outcome. Intact cognition was set as a reference point.

Boldface means statistically significant association.

APCCD, asymptomatic post-COVID cognitive disturbances (no persistent symptoms but pathologic NPS tests); SPCCS, subjective persistent COVID-related cognitive sequelae (persistent symptoms but normal NPS tests); PCCD, persistent COVID-related cognitive disturbances (persistent symptoms and pathologic NPS test); Ham-A, Hamilton anxiety scale; Ham-D, Hamilton depression scale; K10, Kessler Psychological Distress scale; Severity, Seven-category Ordinal Scale.

## Discussion

This single center study on a relatively large group of subjects investigated post-COVID-19 neuro-cognitive status both indirectly, through a self-report structured interview, and directly through a battery of neuropsychological tests specially selected to obtain a rapid but broad assessment across multiple cognitive domains.

Interviewed on average three months after the onset of the first symptoms of COVID-19, 191 subjects (47%) attributed to this illness the worsening of previous or the new onset of neurological symptoms including sleep (31.8%), attention (30.3%), and memory (22.4%) disorders. These findings are consistent with several previous studies (Raman et al., 2021; Gasnier et al., 2022) and the well-established notion that COVID-19 leaves behind a burden of persistent symptoms (Nalbandian et al., 2021).

When directly tested with the neuropsychological battery, 20.7% of the subjects had pathologic results. Specifically, 26.6%, 18.7% and 10.9% of subjects obtained either 0 or 1 equivalent scores on Trial Making, Digit Span Backwards and Frontal Assessment Battery tests respectively, showing impairment in selective and divided attention, working memory, short-term memory and executive functions. This was an unanticipated result in consideration of the relatively young age of the population examined and the good average level of education. These data expand the preliminary knowledge acquired in two previous studies with evidence of attention deficit (Zhou et al., 2020), visuo-perception, naming and fluency (Amalakanti et al., 2021).

The pathological performances we observed could be traced back to the so called dual-tasks that are thought to involve prefrontal and frontal subcortical circuits (Worringer et al., 2019) and can be evaluated with tests such as the Trial Making test. Patients perceive these difficulties as a kind of attentional exhaustion, a difficulty in managing multiple tasks simultaneously, the feeling of not being as performing as before and having to exercise greater attention in tasks previously considered simple and almost automatic.

Interesting results also emerged from the regression study. Some characteristics of the study subjects were found to be associated with the probability of being classified into the SPCCS and PCCD groups, in contrast, no significant associations were found for the APCCD group.

A strong association emerged between the history of cardiovascular disease and SPCCS and PCCD groups. In our sample 151 subjects (37.2%) presented with cardiovascular conditions including chronic heart disease, atrial fibrillation, heart failure, stroke, hypertension and diabetes mellitus; of these 53 had SPCCS and 27 had PCCD. The importance of cardiovascular comorbidities in the acute phase of coronavirus infection is well known (Mazza et al., 2020), however the effect on mid- to long-term sequelae is still not well clarified. Intriguingly, these findings may support a line of research linking microvascular injury to the consequences of COVID-19. Indeed, many microvascular alterations have been observed during the acute phase, including endothelial disfunction and injury, microangiopathy, capillary occlusion by thrombosis or activated immune cells and capillary angiogenesis (Ackermann et al., 2020). It is postulated that, even in the absence of decreased blood flow, these changes could be able alone to cause such a radical redistribution of end organ oxygen delivery to be responsible for organ disfunction and failure that may also show up as cognitive and mood disorders. Pre-existing cardiovascular disease may indicate an already altered microcirculation on which COVID-19 played a worsening role (Østergaard, 2021).

Another strong association was found with fatigue. Fatigue is frequent in infections, inflammatory diseases, cancers and neurodegenerative states. It is interpreted as part of the sickness behavior response, a highly conserved biological mechanism in the animal world that can exert a protective effect during the acute phase of diseases (Omdal, 2020). It is the symptom with the highest prevalence in post-Covid and this is also confirmed in our sample where it was reported in 57% of cases. The association between cognitive impairment and fatigue is well documented in COVID-19 (Ceban et al., 2022) and is supported by multiple possible biological mechanisms including cerebral microvascular injury (Lee et al., 2021), hypometabolism in areas associated with motivation (Guedj et al., 2021), endothelial dysfunction (Libby and Lüscher, 2020), hyperinflammation, autoimmunity, latent viral reactivation, multi-organ pathology, and autonomic nervous system dysfunction (Yong, 2021).

An association was found between anxiety symptoms, mental stress, and the SPCCS group. It is sadly well known that the COVID-19 experience, particularly in the time before the discovery of the vaccine and its distribution, was particularly traumatic for affected individuals regardless of the severity of the acute phase (Janiri et al., 2021). An important component of post-COVID-19 cognitive difficulties should therefore be traced to mental status particularly in subjects with good neuropsychological test scores. Treatments aimed at controlling and improving anxiety and stress should probably be implemented in this group.

Female sex and older age were found to be associated with a higher likelihood of being in the PCCD group. Indeed, differences emerged in the performance of key neuropsychological tests according to age and sex. While, it is easily conceivable how age could show as an important determinant, however, it is not clearly evident how sex might be involved in these differences and this could be a topic for further investigation. Regardless of the reasons, it might be important to consider females and older people at risk of developing a post-COVID-19 cognitive disorder with pathologic neuropsychological testing and reserve targeted treatment for them.

A separate comment should be reserved for sleep disorders that were traced to COVID-19 by 32% of the subject. Moreover, when investigated with quantitative instruments such as the PSQI they gave an even worse picture being present in 64.5% of the subjects. Indeed, the probability of receiving a diagnosis of insomnia in the first three months of recovery is known to be higher in patients diagnosed with COVID-19 than in other conditions and is considered compatible with an anticipated alteration in circadian rhythms operated by the virus (Ray and Reddy, 2020; Taquet et al., 2021). As sleep is an integral part of a person's overall and cognitive well-being, in consideration of these data, we cannot but emphasize the need to aggressively investigate and address sleep disorders in the post-COVID-19.

This study had many methodological limitations due to the design and circumstances under which it was conducted i.e., a “real world” post-COVID-19 clinical practice. It's a single center study with no control group or longitudinal follow-up. In addition, after an initial phase in which people were contacted from the hospital's patient lists, later people from the local area began to request to be followed at our center. Therefore, it is not possible to exclude that the sample is enriched with people with a greater burden of disease. Importantly no pre-morbid neuropsychological evaluation was available. This was the main reason why we sought to model the cognitive status by including self-report information along with test performance, indeed the sole use of neuropsychological tests could have inaccurately estimated the problem since an unknown proportion of subjects could have presented pathological performance on tests regardless of COVID-19. Assuming that this difficulty might be widely spread in clinical practice, such approach could be useful to overcome this limitation in future similar studies. Lastly, the data presented in this study are based on assessments conducted in the pre-vaccination era.

The combination of these limitations did not allow us to corroborate with clinical data several hypotheses currently under investigation in this field of research. For example, the contribution of persistent neuroinflammation such as that observed in the so-called chemo-fog (Fernández-Castañeda et al., 2022); or the fact that COVID-19 may bring out neurocognitive and motor disorders already present in a subclinical form (Fearon and Fasano, 2021; Palermo G. et al., 2023; Palermo S. et al., 2023). Further investigation is therefore essential to get a clearer idea of this extremely articulate matter.

## Conclusion

COVID-19 is capable of eliciting persistent neurocognitive alterations which are measurable with widely available test batteries and seem particularly relevant in the areas of executive functions, attention, and working memory. These neurocognitive disorders appear to be associated with a number of factors such as cardiovascular disease history, persistent fatigue, female sex, age, anxiety, and mental health stress. In the context of this ongoing pandemic, it is imperative to intensify and expand research in the field to facilitate the effective identification of those at risk, promote prompt diagnosis of these disorders, and provide timely treatment.

## Data availability statement

The raw data supporting the conclusions of this article will be made available by the authors, without undue reservation.

## Ethics statement

The studies involving human participants were reviewed and approved by Università Cattolica del Sacro Cuore, Roma. The patients/participants provided their written informed consent to participate in this study.

## Author contributions

AL: conceptualization, resources, investigation, methodology, data curation, and writing—original draft. AC: conceptualization, data curation, formal analysis, methodology, software, visualization, and writing—original draft. FB, GB, FC, SR, ER, AS, LS, MTr, AD, and CP: resources and investigation. MTo: project administration, resources, and investigation. DJ: investigation, methodology, and writing—review and editing. GS: project administration, methodology, and supervision. RL and FP: project administration, resources, investigation, and supervision. MF and RB: project administration and supervision. FL: conceptualization, project administration, resources, supervision, data curation, and writing—review and editing. AB: conceptualization, resources, investigation, methodology, data curation, supervision, and writing—original draft. All authors contributed to the article and approved the submitted version.
