# Supplementary material for: Neuropsychological measures of post-COVID-19 cognitive status
Source: Front Psychol. 2023 Jul 10;14:1136667. doi: 10.3389/fpsyg.2023.1136667 (PMC10363721; doi:10.3389/fpsyg.2023.1136667)
Supplement: Supplementary file 1 [file Data_Sheet_1.docx]

Supplementary materials for “Neuropsychological measures of post-Covid-19 cognitive status”

Carfì A.

Gemelli Against COVID-19 Post-acute Care Study Group, Fondazione Policlinico Universitario Agostino Gemelli IRCCS, Rome, Italy.

Summary

[Table 1. Sample description 2](#_Toc123635172)

[Table 2 Post Covid-19 neurological symptoms self-report 4](#_Toc123635173)

[Table 3. Neuropsychological tests 5](#_Toc123635174)

[Table 4. Variables coding 7](#_Toc123635175)

[Logistic regression and approach to missing data 8](#_Toc123635176)

[Figure 1. Neurological symptoms during acute and post-Covid-19 9](#_Toc123635177)

[Figure 2 – Self reported Covid-related neurological symptoms 10](#_Toc123635178)

[Figure 3 – Neuropsychological tests 11](#_Toc123635179)

[Bibliorgaphy 12](#_Toc123635180)

# Table 1. Sample description

|  |  | **Sex** | | | **Cognitive status** | | | | |
| --- | --- | --- | --- | --- | --- | --- | --- | --- | --- |
|  | **Total** | **Males** | **Females** | **p** | **Intact** | **Symptomatic Persistent Covid-related Cognitive Sequelae (SPCCS)** | **Asymptomatic Post-Covid Cognitive Disturbances (APCCD)** | **Persistent Covid-related Cognitive Disturbances (PCCD)** | **p** |
|  | n = 406 | n = 223 | n = 183 |  | n = 181 | n = 141 | n = 34 | n = 50 |  |
| **General information** |  |  |  |  |  |  |  |  |  |
| Age (years) | 54.5 (15.1) | 57.0 (14.3) | 51.4 (15.6) | <.001 | 53.8 (15.0) | 51.8 (14.1) | 59.5 (15.8) | 61.0 (15.8) | <.001 |
| Females | 183 (45.1%) |  |  |  | 65 (35.9%) | 77 (54.6%) | 13 (38.2%) | 28 (56%) | 0.002 |
| BMI (Kg/m^2^) | 26.0 (4.5) | 26.5 (3.9) | 25.3 (5.1) | 0.007 | 25.7 (4.3) | 26.2 (5.0) | 26.0 (3.6) | 26.4 (4.7) | 0.632 |
| Education years | 14.5 (5.4) | 14.5 (4.5) | 14.6 (6.3) | 0.802 | 15.1 (4.0) | 14.4 (3.7) | 14.9 (12.5) | 12.4 (5.1) | 0.009 |
| Not employed | 137 (33.7%) | 68 (30.5%) | 69 (37.7%) | 0.155 | 55 (30.4%) | 42 (29.8%) | 16 (47.1%) | 24 (48%) | 0.027 |
| Flu vaccination | 97 (23.9%) | 59 (26.5%) | 38 (20.8%) | 0.225 | 35 (19.3%) | 37 (26.2%) | 11 (32.4%) | 14 (28%) | 0.198 |
| Anti-pneumococcal vaccination | 33 (8.1%) | 23 (10.3%) | 10 (5.5%) | 0.108 | 10 (5.5%) | 13 (9.2%) | 6 (17.6%) | 4 (8%) | 0.113 |
| Regular physical activity | 239 (58.9%) | 136 (61%) | 103 (56.3%) | 0.237 | 118 (65.2%) | 76 (53.9%) | 20 (58.8%) | 25 (50%) | 0.082 |
| Smoking status |  |  |  | 0.006 |  |  |  |  | 0.477 |
| Non smoker | 197 (48.5%) | 93 (41.7%) | 104 (56.8%) |  | 86 (47.5%) | 66 (46.8%) | 16 (47.1%) | 29 (58%) |  |
| Active smoker | 29 (7.1%) | 16 (7.2%) | 13 (7.1%) |  | 13 (7.2%) | 7 (5%) | 3 (8.8%) | 6 (12%) |  |
| Former smoker | 152 (37.4%) | 98 (43.9%) | 54 (29.5%) |  | 71 (39.2%) | 55 (39%) | 13 (38.2%) | 13 (26%) |  |
| Unknown | 28 (6.9%) | 16 (7.2%) | 12 (6.6%) |  | 11 (6.1%) | 13 (9.2%) | 2 (5.9%) | 2 (4%) |  |
| **Pre-COVID clinical features** |  |  |  |  |  |  |  |  |  |
| Cardiovascular conditions | 151 (37.2%) | 103 (46.2%) | 48 (26.2%) | <.001 | 56 (30.9%) | 53 (37.6%) | 15 (44.1%) | 27 (54%) | 0.02 |
| Chronic heart disease | 28 (6.9%) | 23 (10.3%) | 5 (2.7%) | 0.005 | 10 (5.5%) | 7 (5%) | 3 (8.8%) | 8 (16%) | 0.046 |
| Atrial fibrillation | 16 (3.9%) | 10 (4.5%) | 6 (3.3%) | 0.715 | 6 (3.3%) | 5 (3.5%) | 3 (8.8%) | 2 (4%) | 0.496 |
| Heart failure | 9 (2.2%) | 7 (3.1%) | 2 (1.1%) | 0.292 | 3 (1.7%) | 4 (2.8%) | 1 (2.9%) | 1 (2%) | 0.895 |
| Stroke | 3 (0.7%) | 1 (0.4%) | 2 (1.1%) | 0.863 | 0 (0%) | 1 (0.7%) | 1 (2.9%) | 1 (2%) | 0.197 |
| Hypertension | 129 (31.8%) | 89 (39.9%) | 40 (21.9%) | <.001 | 49 (27.1%) | 46 (32.6%) | 11 (32.4%) | 23 (46%) | 0.087 |
| Diabetes mellitus | 33 (8.1%) | 24 (10.8%) | 9 (4.9%) | 0.05 | 13 (7.2%) | 10 (7.1%) | 2 (5.9%) | 8 (16%) | 0.187 |
| Renal failure | 12 (3%) | 8 (3.6%) | 4 (2.2%) | 0.592 | 2 (1.1%) | 7 (5%) | 0 (0%) | 3 (6%) | 0.079 |
| Thyroid disease | 66 (16.3%) | 18 (8.1%) | 48 (26.2%) | <.001 | 28 (15.5%) | 27 (19.1%) | 3 (8.8%) | 8 (16%) | 0.507 |
| COPD | 33 (8.1%) | 21 (9.4%) | 12 (6.6%) | 0.386 | 10 (5.5%) | 12 (8.5%) | 5 (14.7%) | 6 (12%) | 0.2 |
| Active cancer | 10 (2.5%) | 7 (3.1%) | 3 (1.6%) | 0.517 | 6 (3.3%) | 3 (2.1%) | 0 (0%) | 1 (2%) | 0.679 |
| Immune disease | 34 (8.4%) | 15 (6.7%) | 19 (10.4%) | 0.253 | 17 (9.4%) | 14 (9.9%) | 1 (2.9%) | 2 (4%) | 0.356 |
| **COVID-19 events** | 92 (22.7%) | 51 (22.9%) | 41 (22.4%) | 1 | 39 (21.5%) | 34 (24.1%) | 10 (29.4%) | 9 (18%) | 0.615 |
| *Seven category ordinal scale* |  |  |  | <.001 |  |  |  |  | 0.345 |
| 2. not hospitalized | 127 (31.3%) | 44 (19.7%) | 83 (45.4%) |  | 50 (27.6%) | 52 (36.9%) | 7 (20.6%) | 18 (36%) |  |
| 3. hosp., not requiring O_2_ | 88 (21.7%) | 41 (18.4%) | 47 (25.7%) |  | 47 (26%) | 28 (19.9%) | 8 (23.5%) | 5 (10%) |  |
| 4. hosp, requiring O_2_ | 125 (30.8%) | 86 (38.6%) | 39 (21.3%) |  | 55 (30.4%) | 40 (28.4%) | 13 (38.2%) | 17 (34%) |  |
| 5. hosp, requiring HFNC/NIV | 34 (8.4%) | 28 (12.6%) | 6 (3.3%) |  | 18 (9.9%) | 10 (7.1%) | 2 (5.9%) | 4 (8%) |  |
| 6. hosp, requiring intubation/ECMO | 32 (7.9%) | 24 (10.8%) | 8 (4.4%) |  | 11 (6.1%) | 11 (7.8%) | 4 (11.8%) | 6 (12%) |  |
| *Drug treatments* |  |  |  |  |  |  |  |  |  |
| Treatment for Covid-19 pneumonia | 231 (56.9%) | 155 (69.5%) | 76 (41.5%) | <.001 | 116 (64.1%) | 66 (46.8%) | 24 (70.6%) | 25 (50%) | 0.004 |
| Anti retro-virals | 209 (51.5%) | 144 (64.6%) | 65 (35.5%) | <.001 | 108 (59.7%) | 60 (42.6%) | 19 (55.9%) | 22 (44%) | 0.013 |
| Hydroxychloroquine | 235 (57.9%) | 152 (68.2%) | 83 (45.4%) | <.001 | 117 (64.6%) | 66 (46.8%) | 26 (76.5%) | 26 (52%) | 0.001 |
| Anti-IL6 | 107 (26.4%) | 82 (36.8%) | 25 (13.7%) | <.001 | 51 (28.2%) | 35 (24.8%) | 8 (23.5%) | 13 (26%) | 0.876 |
| Azythromycin | 144 (35.5%) | 88 (39.5%) | 56 (30.6%) | 0.08 | 74 (40.9%) | 36 (25.5%) | 14 (41.2%) | 20 (40%) | 0.025 |
| Other antibiotics | 125 (30.8%) | 88 (39.5%) | 37 (20.2%) | <.001 | 53 (29.3%) | 46 (32.6%) | 14 (41.2%) | 12 (24%) | 0.359 |
| Enoxaparin | 197 (48.5%) | 128 (57.4%) | 69 (37.7%) | <.001 | 87 (48.1%) | 64 (45.4%) | 22 (64.7%) | 24 (48%) | 0.247 |
| Corticosteroids | 52 (12.8%) | 26 (11.7%) | 26 (14.2%) | 0.538 | 19 (10.5%) | 24 (17%) | 3 (8.8%) | 6 (12%) | 0.306 |
| Anti-platelet drugs | 30 (7.4%) | 23 (10.3%) | 7 (3.8%) | 0.022 | 10 (5.5%) | 8 (5.7%) | 5 (14.7%) | 7 (14%) | 0.061 |
| Length of stay (days) | 17.9 (14.9) | 20.2 (15.9) | 13.9 (12.2) | <.001 | 17.2 (13.8) | 17.7 (15.3) | 15.5 (12.5) | 23.2 (19.3) | 0.143 |
| **Post COVID-19** |  |  |  |  |  |  |  |  |  |
| Days since first symptoms | 97.8 (48.0) | 93.1 (44.3) | 103.5 (51.7) | 0.031 | 87.8 (40.2) | 105.9 (54.2) | 95.5 (42.0) | 112.5 (52.7) | 0.001 |
| Days since hospital discharge | 64.3 (40.3) | 60.9 (37.9) | 70.0 (43.7) | 0.059 | 54.8 (33.0) | 73.8 (44.8) | 66.6 (37.5) | 73.6 (48.6) | 0.003 |
| N. persistent symptoms | 3.2 (2.8) | 2.5 (2.2) | 4.1 (3.3) | <.001 | 2.4 (2.2) | 4.3 (3.3) | 2.6 (2.0) | 3.7 (2.7) | <.001 |
| Persistent symptoms |  |  |  | <.001 |  |  |  |  | <.001 |
| No symptoms | 66 (16.3%) | 47 (21.1%) | 19 (10.4%) |  | 42 (23.2%) | 15 (10.6%) | 4 (11.8%) | 5 (10%) |  |
| 1-2 symptoms | 132 (32.5%) | 81 (36.3%) | 51 (27.9%) |  | 68 (37.6%) | 38 (27%) | 14 (41.2%) | 12 (24%) |  |
| Three or more symptoms | 208 (51.2%) | 95 (42.6%) | 113 (61.7%) |  | 71 (39.2%) | 88 (62.4%) | 16 (47.1%) | 33 (66%) |  |
| Drop in QoL (EQ-vas) | -13.0 (17.3) | -10.7 (14.1) | -15.8 (20.3) | 0.006 | -8.8 (13.1) | -17.1 (19.1) | -11.1 (12.9) | -17.6 (23.0) | <.001 |

BMI: Body Mass Index; COPD: Chronic Obstructive Pulmonary Disease; HFNC: High Flow Nasal Cannulae; NIV: Non-Invasive Ventilation; ECMO: Extra Corporeal Membrane Oxygenation; QoL: Quality of Life; EQ-vas: EuroQol visual analog scale

# Table 2 Post Covid-19 neurological symptoms self-report

| **n = 406** | **Sleep** | **Attention** | **Memory** | **Language** | **Balance** | **LOC** |
| --- | --- | --- | --- | --- | --- | --- |
| No symptoms | 181 (44.5%) | 205 (50.4%) | 267 (65.8%) | 345 (84.9%) | 336 (82.7%) | 366 (90.1%) |
| Pre-existent unaltered† | 21 (5.2%) | 17 (4.2%) | 25 (6.2%) | 5 (1.2%) | 3 (0.7%) | 0 (0%) |
| Covid-19-related subsided | 75 (18.5%) | 61 (15%) | 23 (5.7%) | 16 (3.9%) | 46 (11.3%) | 39 (9.6%) |
| Pre-existent worsened*† | 28 (6.9%) | 10 (2.5%) | 14 (3.4%) | 10 (2.5%) | 3 (0.7%) | 0 (0%) |
| Covid-19-related persistent* | 91 (22.4%) | 101 (24.9%) | 67 (16.5%) | 27 (6.7%) | 15 (3.7%) | 0 (0%) |
| Covid-19-related late-onset* | 10 (2.5%) | 12 (3%) | 10 (2.5%) | 3 (0.7%) | 3 (0.7%) | 1 (0.2%) |
|  |  |  |  |  |  |  |
| †Pre-existent at Covid-19  **n = 88 (21.7%)** | 49 (12.1%) | 27 (6.7%) | 39 (9.6%) | 15 (3.7%) | 6 (1.5%) | 0 |
| *PCNS  **n = 191 (47%)** | 129 (31.8%) | 123 (30.3%) | 91 (22.4%) | 40 (9.9%) | 21 (5.2%) | 1 (0.2%) |

LOC: Loss of consciousness; PCNS: Persistent Covid-related Neurological Symptoms

# Table 3. Neuropsychological tests

|  |  | **Sex** |  |  | **Age grps.** |  |  |  | **Post Covid-19 Cognitive Status** |  |  |  |  | **Seven Category Ordinal Scale** |  |  |  |  |  |
| --- | --- | --- | --- | --- | --- | --- | --- | --- | --- | --- | --- | --- | --- | --- | --- | --- | --- | --- | --- |
|  | **Total** | **Males** | **Females** | **p** | **Under 44** | **45-64** | **65 or more** | **p** | **Intact** | **SPCCS** | **APCCD** | **PCCD** | **p** | **2.No_hosp.** | **3.Hosp.No-O2** | **4.Hosp.O2** | **5.Hosp.HF/NIV** | **6.Hosp.Tube/ECMO** | **p** |
|  | n = 406 | n = 223 | n = 183 |  | n = 93 | n = 213 | n = 100 |  | n = 181 | n = 141 | n = 34 | n = 50 |  | n = 127 | n = 88 | n = 125 | n = 34 | n = 32 |  |
| **Mini Mental State Exam** |  |  |  |  |  |  |  |  |  |  |  |  |  |  |  |  |  |  |  |
| raw | 28.9 (1.4) | 29.1 (1.3) | 28.8 (1.5) | 0.072 | 29.4 (1.2) | 29.1 (1.1) | 28.2 (1.7) | <.001 | 29.3 (0.9) | 29.1 (1.0) | 27.8 (2.1) | 27.8 (2.0) | <.001 | 29.1 (1.4) | 29.2 (1.0) | 28.6 (1.7) | 28.9 (1.2) | 28.9 (0.9) | 0.019 |
| corrected | 28.6 (1.6) | 28.7 (1.5) | 28.4 (1.7) | 0.028 | 28.9 (1.8) | 28.6 (1.6) | 28.3 (1.5) | 0.027 | 29.0 (1.3) | 28.6 (1.4) | 27.5 (1.8) | 27.7 (2.2) | <.001 | 28.5 (1.8) | 28.8 (1.4) | 28.5 (1.7) | 28.6 (1.4) | 28.6 (1.2) | 0.628 |
| **Rey’s immediate recall** |  |  |  |  |  |  |  |  |  |  |  |  |  |  |  |  |  |  |  |
| raw | 47.9 (10.9) | 46.8 (11.5) | 49.1 (10.0) | 0.032 | 56.0 (7.2) | 48.9 (9.2) | 38.0 (9.5) | <.001 | 50.6 (9.2) | 49.4 (9.6) | 39.3 (11.6) | 39.3 (12.2) | <.001 | 51.1 (10.4) | 51.1 (10.0) | 43.4 (10.5) | 45.8 (10.7) | 46.0 (10.1) | <.001 |
| corrected | 45.5 (8.3) | 45.1 (8.8) | 45.9 (7.6) | 0.346 | 47.1 (7.2) | 46.1 (8.6) | 42.6 (7.8) | <.001 | 47.5 (7.0) | 46.3 (7.7) | 39.0 (8.0) | 40.1 (9.8) | <.001 | 46.1 (8.3) | 47.1 (8.2) | 43.2 (7.9) | 45.7 (8.5) | 46.9 (8.3) | 0.006 |
| equivalent | 3.5 (1.0) | 3.4 (1.1) | 3.6 (0.9) | 0.031 | 3.7 (0.7) | 3.5 (1.0) | 3.1 (1.1) | <.001 | 3.7 (0.7) | 3.5 (0.8) | 2.7 (1.4) | 2.8 (1.4) | <.001 | 3.5 (1.0) | 3.6 (0.8) | 3.3 (1.1) | 3.4 (0.9) | 3.5 (0.9) | 0.125 |
| **Rey’s delayed recall** |  |  |  |  |  |  |  |  |  |  |  |  |  |  |  |  |  |  |  |
| raw | 10.0 (3.1) | 9.5 (3.3) | 10.6 (2.8) | <.001 | 12.4 (1.9) | 10.3 (2.5) | 7.2 (3.2) | <.001 | 10.8 (2.7) | 10.4 (2.7) | 7.8 (3.3) | 7.6 (3.9) | <.001 | 11.2 (2.8) | 10.7 (2.8) | 8.7 (3.1) | 9.1 (3.3) | 9.5 (3.0) | <.001 |
| corrected | 9.5 (2.7) | 9.2 (2.8) | 9.9 (2.4) | 0.011 | 10.2 (2.7) | 9.6 (2.4) | 8.7 (2.9) | <.001 | 10.2 (2.4) | 9.6 (2.3) | 7.8 (2.3) | 7.8 (3.4) | <.001 | 10.1 (2.7) | 9.8 (2.6) | 8.7 (2.5) | 9.3 (2.7) | 9.9 (2.6) | <.001 |
| equivalent | 3.3 (1.0) | 3.2 (1.1) | 3.5 (0.9) | <.001 | 3.6 (0.7) | 3.4 (0.9) | 3.0 (1.3) | <.001 | 3.6 (0.7) | 3.4 (0.8) | 2.7 (1.2) | 2.6 (1.6) | <.001 | 3.5 (0.9) | 3.4 (1.0) | 3.1 (1.1) | 3.2 (1.1) | 3.4 (1.0) | 0.025 |
| **Multiple Feature Target Cancellation Test** |  |  |  |  |  |  |  |  |  |  |  |  |  |  |  |  |  |  |  |
| *time* raw | 53.8 (23.7) | 52.7 (21.9) | 55.1 (25.6) | 0.303 | 46.9 (17.1) | 50.5 (17.8) | 67.0 (33.2) | <.001 | 50.6 (19.7) | 49.6 (14.7) | 72.0 (40.8) | 64.5 (31.9) | <.001 | 49.8 (19.2) | 54.2 (24.7) | 56.3 (26.1) | 52.0 (20.6) | 60.2 (27.8) | 0.108 |
| *time* corrected | 53.8 (22.6) | 56.3 (20.6) | 50.8 (24.5) | 0.015 | 59.4 (19.0) | 52.8 (18.1) | 50.8 (31.7) | 0.01 | 53.5 (18.1) | 50.4 (16.8) | 68.1 (38.9) | 54.8 (31.6) | 0.047 | 53.7 (19.0) | 56.9 (21.5) | 52.4 (26.3) | 49.4 (20.8) | 55.9 (24.5) | 0.462 |
| *time* equivalent | 3.9 (0.5) | 3.9 (0.5) | 3.9 (0.6) | 0.919 | 3.8 (0.6) | 3.9 (0.4) | 3.8 (0.8) | 0.043 | 3.9 (0.4) | 4.0 (0.3) | 3.6 (1.0) | 3.6 (0.9) | 0.028 | 3.9 (0.5) | 3.8 (0.6) | 3.9 (0.5) | 3.9 (0.2) | 3.9 (0.7) | 0.88 |
| *correct answers* | 11.6 (1.6) | 11.7 (1.4) | 11.4 (1.8) | 0.15 | 11.8 (1.8) | 11.6 (1.5) | 11.2 (1.7) | 0.029 | 11.9 (1.2) | 11.8 (1.4) | 10.5 (1.9) | 10.6 (2.4) | <.001 | 11.6 (1.8) | 11.8 (1.4) | 11.4 (1.6) | 11.6 (1.3) | 11.5 (1.4) | 0.547 |
| *false alarms* raw | 0.5 (1.8) | 0.5 (2.1) | 0.4 (1.2) | 0.889 | 0.4 (1.3) | 0.4 (1.5) | 0.7 (2.5) | 0.362 | 0.1 (0.4) | 0.2 (0.8) | 1.4 (4.0) | 1.6 (3.2) | 0.001 | 0.6 (2.4) | 0.3 (0.9) | 0.4 (1.6) | 0.2 (0.7) | 0.6 (2.0) | 0.621 |
| *false alarms* corrected | 0.5 (1.8) | 0.5 (2.1) | 0.5 (1.3) | 0.955 | 0.4 (1.5) | 0.4 (1.5) | 0.7 (2.5) | 0.54 | 0.1 (0.4) | 0.3 (0.8) | 1.5 (3.9) | 1.7 (3.3) | 0.001 | 0.7 (2.4) | 0.3 (1.0) | 0.4 (1.7) | 0.3 (0.7) | 0.6 (2.1) | 0.558 |
| *false alarms* equivalent | 3.5 (1.1) | 3.6 (1.0) | 3.5 (1.1) | 0.098 | 3.5 (1.0) | 3.6 (1.0) | 3.5 (1.1) | 0.881 | 3.8 (0.6) | 3.7 (0.9) | 2.9 (1.4) | 2.7 (1.7) | <.001 | 3.4 (1.2) | 3.6 (0.9) | 3.6 (0.9) | 3.6 (0.9) | 3.5 (1.2) | 0.366 |
| *accuracy* | 0.9 (0.1) | 0.9 (0.1) | 0.9 (0.1) | 0.176 | 1.0 (0.1) | 0.9 (0.1) | 0.9 (0.1) | 0.022 | 1.0 (0.0) | 0.9 (0.1) | 0.9 (0.1) | 0.9 (0.1) | <.001 | 0.9 (0.1) | 0.9 (0.1) | 0.9 (0.1) | 0.9 (0.0) | 0.9 (0.1) | 0.532 |
| **Frontal Assessment Battery** |  |  |  |  |  |  |  |  |  |  |  |  |  |  |  |  |  |  |  |
| raw | 16.5 (1.7) | 16.5 (1.7) | 16.6 (1.6) | 0.462 | 17.2 (0.8) | 16.7 (1.4) | 15.4 (2.2) | <.001 | 17.0 (1.0) | 16.9 (1.1) | 15.0 (2.5) | 14.9 (2.4) | <.001 | 16.9 (1.4) | 16.9 (1.3) | 16.1 (2.0) | 16.4 (1.2) | 15.9 (2.1) | <.001 |
| corrected | 16.1 (1.6) | 16.2 (1.7) | 16.1 (1.6) | 0.958 | 16.4 (1.4) | 16.2 (1.6) | 15.8 (1.9) | 0.059 | 16.5 (1.2) | 16.5 (1.3) | 14.7 (2.0) | 14.9 (2.3) | <.001 | 16.4 (1.5) | 16.3 (1.4) | 16.0 (1.9) | 16.1 (1.1) | 15.8 (2.1) | 0.219 |
| equivalent | 3.0 (1.1) | 3.0 (1.1) | 2.9 (1.1) | 0.43 | 3.1 (1.0) | 3.0 (1.1) | 2.8 (1.4) | 0.299 | 3.3 (0.8) | 3.2 (0.9) | 2.0 (1.4) | 2.0 (1.5) | <.001 | 3.0 (1.1) | 3.1 (0.9) | 2.9 (1.2) | 3.0 (0.9) | 2.7 (1.6) | 0.506 |
| **Digit Span Forward** |  |  |  |  |  |  |  |  |  |  |  |  |  |  |  |  |  |  |  |
| raw | 6.0 (1.2) | 6.1 (1.2) | 5.9 (1.1) | 0.237 | 6.3 (1.1) | 6.0 (1.2) | 5.7 (1.1) | 0.002 | 6.3 (1.1) | 6.2 (1.1) | 4.9 (1.0) | 5.1 (0.9) | <.001 | 6.0 (1.1) | 6.1 (1.3) | 5.9 (1.2) | 6.1 (1.1) | 5.9 (1.3) | 0.479 |
| corrected | 5.8 (1.2) | 6.0 (1.2) | 5.7 (1.2) | 0.047 | 5.8 (1.2) | 5.8 (1.2) | 6.0 (1.1) | 0.359 | 6.1 (1.1) | 6.0 (1.2) | 4.9 (1.1) | 5.2 (1.0) | <.001 | 5.7 (1.0) | 5.9 (1.3) | 5.8 (1.2) | 6.1 (1.0) | 6.0 (1.3) | 0.522 |
| equivalent | 3.1 (1.2) | 3.1 (1.2) | 3.0 (1.2) | 0.177 | 3.0 (1.1) | 3.0 (1.3) | 3.2 (1.3) | 0.555 | 3.4 (0.9) | 3.3 (1.0) | 1.7 (1.7) | 2.3 (1.5) | <.001 | 3.0 (1.1) | 3.0 (1.3) | 3.1 (1.3) | 3.4 (1.2) | 3.0 (1.3) | 0.624 |
| **Digit Span Backwards** |  |  |  |  |  |  |  |  |  |  |  |  |  |  |  |  |  |  |  |
| raw | 4.2 (1.0) | 4.3 (1.1) | 4.1 (0.9) | 0.007 | 4.5 (1.0) | 4.3 (1.0) | 3.8 (1.0) | <.001 | 4.5 (1.0) | 4.4 (0.9) | 3.1 (0.8) | 3.3 (0.8) | <.001 | 4.4 (0.9) | 4.3 (1.0) | 4.0 (1.1) | 4.2 (1.3) | 4.2 (0.9) | 0.022 |
| corrected | 4.0 (1.0) | 4.2 (1.0) | 3.8 (0.9) | <.001 | 3.9 (1.0) | 4.0 (1.0) | 4.1 (1.0) | 0.437 | 4.2 (0.9) | 4.2 (0.9) | 3.1 (0.8) | 3.3 (0.9) | <.001 | 4.0 (0.9) | 4.0 (1.0) | 3.9 (1.1) | 4.2 (1.1) | 4.2 (1.0) | 0.568 |
| equivalent | 2.7 (1.3) | 2.8 (1.2) | 2.4 (1.3) | 0.002 | 2.5 (1.4) | 2.7 (1.2) | 2.7 (1.3) | 0.42 | 3.0 (1.1) | 2.9 (1.2) | 1.4 (1.2) | 1.7 (1.3) | <.001 | 2.7 (1.2) | 2.6 (1.3) | 2.5 (1.3) | 2.8 (1.3) | 2.8 (1.3) | 0.513 |
| **Trail Making** |  |  |  |  |  |  |  |  |  |  |  |  |  |  |  |  |  |  |  |
| raw | 102.3 (70.8) | 96.1 (67.9) | 109.9 (73.8) | 0.051 | 80.4 (52.2) | 92.1 (59.2) | 144.3 (89.6) | <.001 | 83.9 (50.1) | 82.3 (44.4) | 168.5 (91.2) | 180.1 (97.0) | <.001 | 97.6 (70.6) | 84.7 (46.8) | 113.8 (76.8) | 99.4 (77.2) | 127.7 (83.6) | 0.004 |
| corrected | 100.8 (71.4) | 89.6 (68.5) | 114.3 (72.7) | <.001 | 107.8 (50.6) | 89.5 (60.2) | 118.2 (100.6) | 0.003 | 83.2 (51.7) | 81.5 (44.1) | 163.6 (93.6) | 175.7 (101.1) | <.001 | 108.6 (68.2) | 89.6 (48.7) | 101.5 (80.0) | 86.4 (78.3) | 112.2 (89.6) | 0.127 |
| equivalent | 2.5 (1.3) | 2.7 (1.3) | 2.2 (1.3) | <.001 | 2.1 (1.1) | 2.7 (1.2) | 2.3 (1.5) | <.001 | 2.8 (1.1) | 2.7 (1.1) | 1.4 (1.3) | 1.3 (1.5) | <.001 | 2.3 (1.3) | 2.6 (1.1) | 2.5 (1.4) | 2.8 (1.3) | 2.3 (1.4) | 0.153 |
| **Female sex** |  |  |  |  | 58 (62.4%) | 90 (42.3%) | 35 (35%) | <.001 | 65 (35.9%) | 77 (54.6%) | 13 (38.2%) | 28 (56%) | 0.002 | 83 (65.4%) | 47 (53.4%) | 39 (31.2%) | 6 (17.6%) | 8 (25%) | <.001 |
| **Treatment for Covid-19 pneumonia** | 231 (56.9%) | 155 (69.5%) | 76 (41.5%) | <.001 | 29 (31.2%) | 121 (56.8%) | 81 (81%) | <.001 | 116 (64.1%) | 66 (46.8%) | 24 (70.6%) | 25 (50%) | 0.004 | 6 (4.7%) | 58 (65.9%) | 104 (83.2%) | 32 (94.1%) | 31 (96.9%) | <.001 |
| **Post Covid-19 Cognitive Status** |  |  |  | 0.002 |  |  |  | <.001 |  |  |  |  |  |  |  |  |  |  | 0.345 |
| Intact | 181 (44.6%) | 116 (52%) | 65 (35.5%) |  | 42 (45.2%) | 99 (46.5%) | 40 (40%) |  |  |  |  |  |  | 50 (39.4%) | 47 (53.4%) | 55 (44%) | 18 (52.9%) | 11 (34.4%) |  |
| SPCCS | 141 (34.7%) | 64 (28.7%) | 77 (42.1%) |  | 37 (39.8%) | 81 (38%) | 23 (23%) |  |  |  |  |  |  | 52 (40.9%) | 28 (31.8%) | 40 (32%) | 10 (29.4%) | 11 (34.4%) |  |
| APCCD | 34 (8.4%) | 21 (9.4%) | 13 (7.1%) |  | 6 (6.5%) | 14 (6.6%) | 14 (14%) |  |  |  |  |  |  | 7 (5.5%) | 8 (9.1%) | 13 (10.4%) | 2 (5.9%) | 4 (12.5%) |  |
| PCCD | 50 (12.3%) | 22 (9.9%) | 28 (15.3%) |  | 8 (8.6%) | 19 (8.9%) | 23 (23%) |  |  |  |  |  |  | 18 (14.2%) | 5 (5.7%) | 17 (13.6%) | 4 (11.8%) | 6 (18.8%) |  |
| **Age group** |  |  |  | <.001 |  |  |  |  |  |  |  |  | 0.005 |  |  |  |  |  | <.001 |
| Under 44 | 101 (24.9%) | 39 (17.5%) | 62 (33.9%) |  |  |  |  |  | 46 (25.4%) | 41 (29.1%) | 6 (17.6%) | 8 (16%) |  | 54 (42.5%) | 29 (33%) | 16 (12.8%) | 2 (5.9%) | 0 (0%) |  |
| 45-64 | 213 (52.5%) | 123 (55.2%) | 90 (49.2%) |  |  |  |  |  | 98 (54.1%) | 78 (55.3%) | 15 (44.1%) | 22 (44%) |  | 64 (50.4%) | 46 (52.3%) | 68 (54.4%) | 16 (47.1%) | 19 (59.4%) |  |
| 65 or more | 92 (22.7%) | 61 (27.4%) | 31 (16.9%) |  |  |  |  |  | 37 (20.4%) | 22 (15.6%) | 13 (38.2%) | 20 (40%) |  | 9 (7.1%) | 13 (14.8%) | 41 (32.8%) | 16 (47.1%) | 13 (40.6%) |  |
| **Seven category ordinal scale** |  |  |  | <.001 |  |  |  | <.001 |  |  |  |  | 0.345 |  |  |  |  |  |  |
| 2. not hospitalized | 127 (31.3%) | 44 (19.7%) | 83 (45.4%) |  | 52 (55.9%) | 63 (29.6%) | 12 (12%) |  | 50 (27.6%) | 52 (36.9%) | 7 (20.6%) | 18 (36%) |  |  |  |  |  |  |  |
| 3. hosp., not requiring O_2_ | 88 (21.7%) | 41 (18.4%) | 47 (25.7%) |  | 27 (29%) | 47 (22.1%) | 14 (14%) |  | 47 (26%) | 28 (19.9%) | 8 (23.5%) | 5 (10%) |  |  |  |  |  |  |  |
| 4. hosp, requiring O_2_ | 125 (30.8%) | 86 (38.6%) | 39 (21.3%) |  | 13 (14%) | 69 (32.4%) | 43 (43%) |  | 55 (30.4%) | 40 (28.4%) | 13 (38.2%) | 17 (34%) |  |  |  |  |  |  |  |
| 5. hosp, requiring HFNC/NIV | 34 (8.4%) | 28 (12.6%) | 6 (3.3%) |  | 1 (1.1%) | 17 (8%) | 16 (16%) |  | 18 (9.9%) | 10 (7.1%) | 2 (5.9%) | 4 (8%) |  |  |  |  |  |  |  |
| 6. hosp, requiring intubation/ECMO | 32 (7.9%) | 24 (10.8%) | 8 (4.4%) |  | 0 (0%) | 17 (8%) | 15 (15%) |  | 11 (6.1%) | 11 (7.8%) | 4 (11.8%) | 6 (12%) |  |  |  |  |  |  |  |

# Table 4. Variables coding

Some study variables have been coded as in the following table.

| **Variable** | **Condition** |
| --- | --- |
| Treatment for Covid-19 pneumonia | At least two of the following treatments during the acute phase: darunavir_ritonavir, lopinavir_ritonavir, remdesivir, hydroxychloroquine, anti_il6, azythromycin, other anti bacterials |
| Anti retro-virals | Any acute treatment with: darunavir_ritonavir, lopinavir_ritonavir or remdesivir |
| Corticosteroids | Any acute treatment with corticosteroids: dexamethasone, prednisone, methyl-prednisolone |
| Anti-platelet drugs | Any use of anti-platelet drugs: acetylsalicylic acid, clopidogrel |
| N. persistent symptoms | Number of self-reported persistent symptoms on the day of the visit: fatigue, cough, diarrhea, headache, anosmia, dysgeusia, red eyes, low vision, syncope, vertigo, joint pain, skin lesions, sicca syndrome, Raynaud phenomenon, myalgia, dyspnea, chest pain, sore throat, sputum production, rhinitis, lack of appetite |
| N. previous neuro. problems | Number of any self-reported pre-existent problems in the domains of memory, attention, language, balance, sleep or syncope |
| Reports pre-Covid sleep problems | Self-reported pre-existent sleep problems |
| History of any cardiovascular condition | Any history of chronic heart disease, atrial fibrillation, heart failure, stroke, hypertension or diabetes mellitus |
| History of psychiatric disturbances | Have you ever suffered from anxiety, depression, nervous breakdowns, substance addiction? |
| Persistent pain | Any joint, chest or muscle pain |
| Persistently elevated acute phase reactants | any of: PCR > 5; d-dimer > 500 or age*10; ESR > 22 (males) or 29 (females) |
| Drop in QoL (scale 1:10) | Drop in EQ-vas scale between pre-Covid and at the time of evaluation (1:10 scale) |
| Anxiety (Ham_A) | Hamilton anxiety total score > 7 |
| Depression (Ham-D) | Hamilton depression total score > 7 |
| Psychological distress (K10) | Kessler K10 total score > 19 |

# Logistic regression and approach to missing data

Multiple logistic regression was performed to assess the association between the outcome variable PCCD and a number of variables of clinical interest.

Missing values were managed with multiple imputation by chained equations technique using *mice* package from R^1,2^. The imputation algorithm chosen was the random forest provided by the *randomForest* package in order to avoid assumptions on data distribution^3^. Outcome variable PCCD was not taken into account in the imputation process in order to prevent any possible influence of the outcome variable on the imputed missing values. The imputation parameters were set to produce 5 imputed datasets (*m = 5*) with 20 iterations per variable (*maxit = 20*) with random forest method (*method = “rf”*) set to 10 branches (*ntree = 10*). The regression model was run on each imputed dataset and pooled results were presented in the main manuscript. Detailed pooled results are shown in the table.

|  |  | **SPCCS** |  |  |  |  |  |  |  | **APCCD** |  |  |  |  |  |  |  | **PCCD** |  |  |  |  |  |  |  |
| --- | --- | --- | --- | --- | --- | --- | --- | --- | --- | --- | --- | --- | --- | --- | --- | --- | --- | --- | --- | --- | --- | --- | --- | --- | --- |
| **term** | **missing** | **estim** | **ubar** | **b** | **t** | **df** | **riv** | **λ** | **fmi** | **estim** | **ubar** | **b** | **t** | **df** | **riv** | **λ** | **fmi** | **estim** | **ubar** | **b** | **t** | **df** | **riv** | **λ** | **fmi** |
| Age |  | -0,016 | 0,000 | 0,000 | 0,000 | 360,292 | 0,011 | 0,011 | 0,016 | 0,020 | 0,000 | 0,000 | 0,000 | 367,900 | 0,000 | 0,000 | 0,006 | 0,041 | 0,000 | 0,000 | 0,000 | 356,982 | 0,014 | 0,014 | 0,019 |
| Female sex |  | 0,407 | 0,077 | 0,000 | 0,077 | 366,686 | 0,003 | 0,003 | 0,008 | 0,273 | 0,182 | 0,000 | 0,182 | 367,301 | 0,002 | 0,002 | 0,007 | 0,786 | 0,153 | 0,000 | 0,153 | 366,540 | 0,003 | 0,003 | 0,009 |
| Body Mass Index | 2 (0.49%) | 0,034 | 0,001 | 0,000 | 0,001 | 357,676 | 0,013 | 0,013 | 0,019 | 0,016 | 0,002 | 0,000 | 0,002 | 367,958 | 0,000 | 0,000 | 0,006 | 0,032 | 0,002 | 0,000 | 0,002 | 361,137 | 0,010 | 0,010 | 0,015 |
| Active smoker | 28 (6.90%) | -0,708 | 0,295 | 0,008 | 0,304 | 330,846 | 0,031 | 0,030 | 0,036 | 0,577 | 0,512 | 0,002 | 0,514 | 365,104 | 0,005 | 0,005 | 0,011 | 0,663 | 0,359 | 0,001 | 0,360 | 366,478 | 0,003 | 0,003 | 0,009 |
| History of attention and memory problems |  | 0,240 | 0,161 | 0,001 | 0,161 | 366,126 | 0,004 | 0,004 | 0,009 | 0,484 | 0,303 | 0,000 | 0,303 | 367,542 | 0,001 | 0,001 | 0,007 | -0,411 | 0,305 | 0,001 | 0,306 | 366,453 | 0,003 | 0,003 | 0,009 |
| History of any cardiovascular condition |  | 0,816 | 0,101 | 0,001 | 0,102 | 363,929 | 0,007 | 0,007 | 0,012 | 0,197 | 0,209 | 0,000 | 0,209 | 367,613 | 0,001 | 0,001 | 0,006 | 1,039 | 0,184 | 0,000 | 0,185 | 366,560 | 0,003 | 0,003 | 0,008 |
| Severity (7 cat.) |  | -0,034 | 0,014 | 0,000 | 0,014 | 366,607 | 0,003 | 0,003 | 0,008 | 0,080 | 0,031 | 0,000 | 0,031 | 367,800 | 0,001 | 0,001 | 0,006 | -0,098 | 0,027 | 0,000 | 0,027 | 367,705 | 0,001 | 0,001 | 0,006 |
| Persistent fatigue | 6 (1.48%) | 0,727 | 0,075 | 0,002 | 0,077 | 339,328 | 0,026 | 0,025 | 0,031 | 0,194 | 0,168 | 0,002 | 0,170 | 356,364 | 0,014 | 0,014 | 0,020 | 1,168 | 0,169 | 0,003 | 0,172 | 343,744 | 0,023 | 0,023 | 0,028 |
| Anxiety (Ham-A) | 24 (5.91%) | 0,602 | 0,078 | 0,010 | 0,090 | 135,757 | 0,150 | 0,130 | 0,143 | 0,406 | 0,206 | 0,004 | 0,211 | 341,309 | 0,025 | 0,024 | 0,030 | 0,233 | 0,150 | 0,008 | 0,160 | 262,990 | 0,064 | 0,060 | 0,067 |
| Depressive sympt. (Ham-D) | 21 (5.17%) | 0,380 | 0,146 | 0,015 | 0,163 | 167,945 | 0,121 | 0,108 | 0,118 | -0,699 | 0,543 | 0,001 | 0,544 | 367,236 | 0,002 | 0,002 | 0,007 | 0,834 | 0,265 | 0,018 | 0,287 | 231,196 | 0,081 | 0,075 | 0,082 |
| Mental health stress (K10) | 71 (17.49%) | 0,410 | 0,027 | 0,004 | 0,031 | 112,253 | 0,178 | 0,151 | 0,166 | -0,072 | 0,106 | 0,014 | 0,123 | 131,793 | 0,154 | 0,133 | 0,146 | 0,367 | 0,046 | 0,009 | 0,058 | 79,317 | 0,238 | 0,192 | 0,212 |

riv = relative increase in variance due to non response

fmi = fraction of missing information

λ = proportion of the total variance attributable to the missing data

# Figure 1. Neurological symptoms during acute and post-Covid-19

The figure shows percentages of patients presenting with specific neurologic Covid-related symptoms during the acute phase of the disease (left) and at the time of the follow-up visit (right).

# Figure 2 – Self reported Covid-related neurological symptoms

Neurological symptoms specifically assessed during the neurologist interview and described by the patient in relation to time of onset and course relative to Covid-19.

# Figure 3 – Neuropsychological tests

The figure shows, for each neuropsychological test, the proportion of patients with fair (light color), borderline (darker color) or failed (dark color) outcome. Equivalent scores (EQ_scores) were used to rate subjects: those with a score of two or more, one or zero were classified as having normal, borderline or pathologic performance respectively.

Horizontal dashed line indicates the overall prevalence of subject classified as having a pathologic neuropsychological test (i.e., at least 1 EQ score of zero and at least 1 EQ score of one)

MFTC: Multiple Features Target Cancellation test.

# Bibliorgaphy

1. van Buuren, S. Multiple imputation of discrete and continuous data by fully conditional specification. *Stat. Methods Med. Res.* **16**, 219–242 (2007).

2. Buuren, S. van & Groothuis-Oudshoorn, K. mice : Multivariate Imputation by Chained Equations in R. *J. Stat. Softw.* **45**, (2011).

3. Shah, A. D., Bartlett, J. W., Carpenter, J., Nicholas, O. & Hemingway, H. Comparison of Random Forest and Parametric Imputation Models for Imputing Missing Data Using MICE: A CALIBER Study. *Am. J. Epidemiol.* **179**, 764–774 (2014).
